# Platelet CD40L Expression Response to Mixing of pRBCs and Washed Platelets but no Causality Association between Platelet ROS Generation and CD40L Expression: An In Vitro Study

**DOI:** 10.3390/antiox11061108

**Published:** 2022-06-02

**Authors:** Mei-Hua Hu, Tien-Yu Huang, Yu-Ching Chou, Go-Shine Huang

**Affiliations:** 1 Division of Pediatric General Medicine, Department of Pediatrics, Chang Gung Memorial Hospital at LinKou, College of Medicine, Chang Gung University, Taoyuan 33302, Taiwan; emerald@cgmh.org.tw; 2 Graduate Institute of Clinical Medical Sciences, College of Medicine, Chang Gung University, Taoyuan 33302, Taiwan; 3 School of Chinese Medicine, College of Medicine, Chang Gung University, Taoyuan 33302, Taiwan; 4 Division of Gastroenterology, Department of Internal medicine, Tri-service General Hospital, National Defense Medical Center, Taipei 11490, Taiwan; tienyu27@gmail.com; 5 School of Public Health, National Defense Medical Center, Taipei 11490, Taiwan; trishow@mail.ndmctsgh.edu.tw; 6 Department of Anesthesiology, Tri-Service General Hospital, National Defense Medical Center, Taipei 11490, Taiwan

**Keywords:** platelet, reactive oxygen species, CD40L, antioxidant, transfusion reaction

## Abstract

Platelets play a role in transfusion reaction via reactive oxygen species (ROS) generation and CD40 ligand (CD40L) expression. In this study, we aimed to test the hypothesis that the mixing of packed red blood cells (pRBCs) and washed platelets has a causal effect on platelet ROS generation and CD40L expression. Thus, a better understanding of this causality relationship may help interrupt the chain of events and avoid an uncontrollable transfusion reaction. We simulated transfusion in vitro by mixing pRBCs and washed platelets. Donor cross-matched stored pRBCs) from our blood bank and recipient whole blood from patients undergoing coronary artery bypass graft surgery prepared into washed platelets were used. Briefly, donor pRBCs were added to washed recipient platelets to form 1%, 5%, or 10% (*v*/*v*) mixtures. The mixed blood sample was used to determine platelet ROS generation (dichlorofluorescein fluorescence levels) and CD40L expression. The effect of antioxidants (20 mM glutamine and 20 mM dipeptiven) on ROS generation and CD40L expression was also evaluated. Platelet ROS generation was not significantly associated with the mixing of pRBCs and washed platelets (*p* = 0.755), glutamine treatment (*p* = 0.800), or dipeptiven treatment (*p* = 0.711). The expression of CD40L by platelets increased significantly (*p* < 0.001), and no significant difference was noted after treatment with glutamine (*p* = 0.560) or dipeptiven (*p* = 0.618). We observed that the mixing pRBCs and washed platelets had no effect via ROS, whereas CD40L could directly induce transfusion reactions. Furthermore, platelets did not causally express ROS or CD40L after being mixed with pRBCs. Although antioxidants are more accessible than anti-CD40L antibodies, platelet ROS may not serve as a therapeutic target for antioxidants. Nevertheless, CD40L expression may be a valuable therapeutic target for managing transfusion reactions.

## 1. Introduction

Platelets play a critical role in the initial reaction to control bleeds by rapidly forming a plug at sites of vascular injury. In the regulation of thrombosis, hemostasis, immunity and inflammation, platelets aggregate with circulating leukocytes, cytokines, thrombin, inflammatory mediators, and endothelium [1,2]. Platelets cause transfusion reactions via generation of reactive oxygen species (ROS) and CD40 ligand (CD40L) expression [2,3,4,5,6,7]. ROS are short-lived, highly reactive molecules—regarded as toxic products of cellular metabolism—and can function as signaling molecules that regulate many physiological processes [8,9]. Additionally, ROS are closely related to transfusion reactions through neutrophils [10,11], macrophages, and other circulatory cells or plasma components [12,13,14,15,16]. The low levels of intraplatelet ROS are associated with low levels of platelet pro-inflammatory molecules in stored platelets [3]. Therapeutic strategies for ROS aimed at the transfused recipient have also shown to be advantageous [15]. CD40L is a transmembrane molecule of crucial interest in immune cell signaling as it binds to several receptors, including CD40 [6,17,18,19]. Previous reports have revealed that CD40L has the potential to cause transfusion reactions [4,16,18,19,20]. For example, platelets involved in CD40/CD40L complex formation cause transfusion-related acute lung injury [17]

However, whether the transfusion reaction is mediated via ROS and/or CD40L in a causal manner in platelets remains unknown. Therefore, we explored the causality relationships between mixed packed red blood cells (pRBCs) and washed platelets, ROS generation, CD40L expression, and transfusion reactions. We hypothesized that by mixing pRBCs and washed platelets it can be possible to induce a causality effect that will initiate ROS generation and further increase CD40L expression to induce a transfusion reaction within platelets. Hence, in this study, we aimed to evaluate whether mixing pRBCs and washed platelets affects platelet ROS generation and CD40L expression. Our study provides an understanding regarding the causality of platelet-mediated transfusion reactions, thereby highlighting impactful instances where the chain reaction can be interrupted to avoid uncontrolled reactions.

## 2. Methods

### 2.1. Reagents

The 2′,7′-dichlorodihydrofluorescein diacetate (DCFH-DA; Cayman Chemical, Ann Arbor, MI, USA) was used as an indicator of peroxynitrite formation for the detection of ROS. Peroxynitrite is an efficient mediator of oxidation, and following enzymatic or base-catalyzed cleavage of the diacetate groups, it is readily oxidized to a highly fluorescent product, dichlorofluorescein (DCF). The anti-CD40L-FITC antibody (BD, Franklin Lakes, NJ, USA) is a monoclonal antibody directed against CD40L expression on the platelet surface. The anti-CD41a-PE (BD, Franklin Lakes, NJ, USA) is a platelet-specific monoclonal antibody, that recognizes the platelet GPIIb/IIIa complex independent of its activation. Additionally, negative IgG1κ-FITC and IgG1κ-PE antibodies (BD, Franklin Lakes, NJ, USA) were used to examine nonspecific binding as background controls. Platelet wash buffer (20 mM 4-(2-hydroxyethyl)-1-piperazineethanesulfonic acid, 145 mM NaCl, 9 mM Na_2_EDTA, pH 7.4) and HEPES-Tyrode’s buffer (10 mM 4-(2-hydroxyethyl)-1-piperazineethanesulfonic acid, 136.89 mM NaCl, 11.9 mM NaHCO_3_, 1.61 mM KCl, 0.42 mM Na_2_HPO_4_, 1.05 mM MgCl_2_, 5.6 mM glucose, pH 7.4) were prepared in our laboratory. Dipeptiven and glutamine were used as antioxidants. Glutamine (Sigma, St. Louis, MO, USA) is a precursor of glutathione, which is an important antioxidant for preventing damage caused by ROS. Dipeptiven (20% l-alanyl-l-glutamine, Fresenius Kabi Co., Wendorff, Austria) is split endogenously into two amino acids—glutamine and alanine, thus, acting as an endogenous glutamine source to platelets.

### 2.2. Patients and Blood Sample Preparation

This study was approved by the institutional review board of Tri-Service General Hospital (TSGHIRB 1-107-05-015), and informed consent was obtained from all participants before enrollment. The trial was registered at the Chinese Clinical Trial Registry (ChiCTR2100045606). http://www.chictr.org.cn/showproj.aspx?proj=123019. Accessed date: 15 April 2022. Donor cross-matched pRBCs with a hematocrit of 55–60% kept at 2–4 °C were acquired from the blood bank of our hospital. One pRBC sample was used for the blood sample of each recipient. Blood samples of the recipients were obtained from patients scheduled for cardiac surgery before induction of anesthesia. The Rationale for specifically using blood from recipients undergoing cardiac surgery was described in Supplemental Materials (Supplemental Figures S1 and S2). Whole blood (18 mL) of the recipients was collected from an arterial catheter using the two-syringe technique to avoid contamination and anticoagulated with a 1:9 volume of a 3.8% sodium citrate solution. The primary outcome measure was the effect of the mixing of pRBCs and washed platelets on platelet ROS generation and CD40L expression. The secondary outcome measure was the effect of antioxidant treatment on platelet ROS generation and CD40L expression.

### 2.3. Preparation of Washed Platelets

Recipient platelet-rich plasma was obtained by centrifugation of whole blood at 200× *g* for 10 min. The platelet-rich plasma was further centrifuged at 2000× *g* for 10 min, and the resulting pellet was resuspended in platelet wash buffer. The suspension was centrifuged at 2000× *g* for 10 min to obtain washed platelets. The supernatant was discarded; the platelets were further resuspended in HEPES-Tyrode’s buffer; the suspension was adjusted to a final cell count of 150,000–450,000 platelets/μL of corresponding whole blood platelet counts.

### 2.4. Quantification of ROS Generation upon Mixing pRBCs and Washed Platelets, Subsequent to Glutamine or Dipeptiven Treatment of Washed Platelets

**Figure 1:**
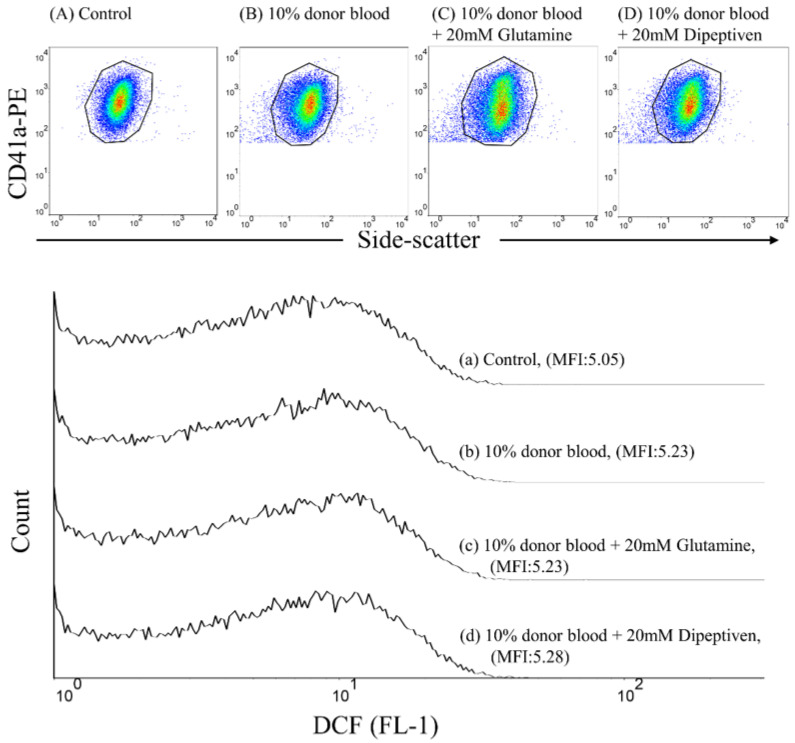
Flow cytometric analysis of DCF fluorescence generation on platelets by 2′,7′-dichlorodihydrofluorescein diacetate (DCFH-DA) staining. Individual platelets were identified by their characteristic side-scatter properties (granularity; *x*-axis) and positive labeling with a platelet-specific monoclonal antibody (CD41a-PE; *y*-axis). Dot plot of the fluorescence of (**A**) control, (**B**) 10% donor blood (*v*/*v*), (**C**) 10% donor blood (*v*/*v*) mixed with 20 mM glutamine, and (**D**) 10% donor blood (*v*/*v*) mixed with 20 mM dipeptiven. IT’S the Overlay of the histograms for (**a**) control, (**b**) 10% donor blood (*v*/*v*), (**c**) 10% donor blood (*v*/*v*) mixed with 20 mM glutamine, and (**d**) 10% donor blood (*v*/*v*) mixed with 20 mM dipeptiven.

Donor pRBCs were added to recipient-washed platelets to form 1%, 5%, or 10% (*v*/*v*) mixtures at 37 °C for 5 min. We also treated 10% (*v*/*v*) mixtures with glutamine (20 mM) or dipeptiven (20 mM) within 1 min to examine whether DCF fluorescence generation can be inhibited at 37 °C for 5 min. FITC-labeled mouse IgG1κ and PE-labeled mouse IgG1κ served as background controls. To quantify ROS generation, the samples were stained with a saturating concentration of the DCFH-DA and anti-CD41a-PE monoclonal antibodies and incubated at approximately 22–28 °C in the dark for 20 min. Individual platelets were identified through anti-CD41a-PE immunofluorescence in a logarithmic-scaled dot plot and side scatter (granularity characteristics). ROS generation was quantified by measuring the increase in DCF fluorescence (Figure 1).

### 2.5. Quantification of CD40L Expression upon Mixing pRBCs and Washed Platelets, Subsequent to Glutamine or Dipeptiven Treatment of Washed Platelets

**Figure 2:**
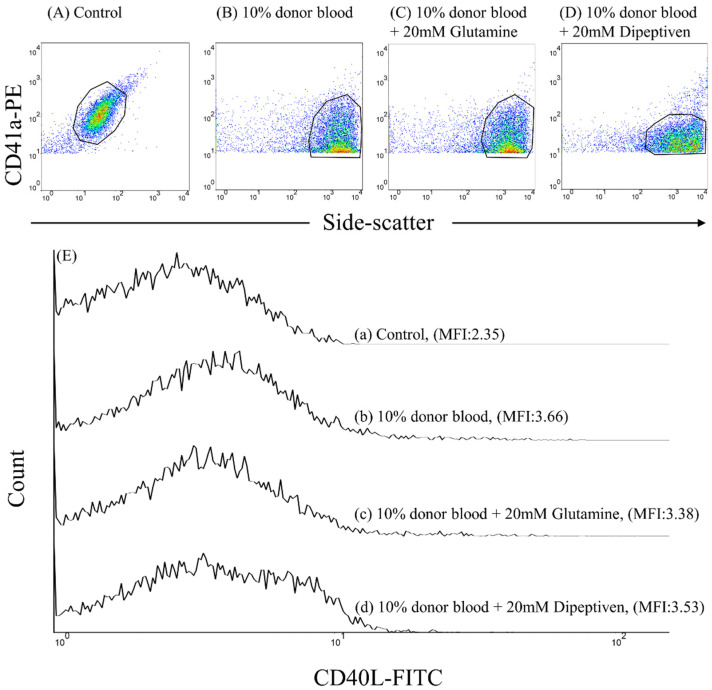
Flow cytometric analysis of platelet CD40L expression. Individual platelets were identified by their characteristic side-scatter properties (granularity; *x*-axis) and positive labeling with a platelet-specific monoclonal antibody (CD41a-PE; *y*-axis). Dot plot of the fluorescence of (**A**) control, (**B**) 10% donor blood (*v*/*v*), (**C**) 10% donor blood (*v*/*v*) mixed with 20 mM glutamine, and (**D**) 10% donor blood (*v*/*v*) mixed with 20 mM dipeptiveit’s the (**E**) Overlay of the histograms for (**a**) control, (**b**) 10% donor blood (*v*/*v*), (**c**) 10% donor blood (*v*/*v*) mixed with 20 mM glutamine, and (**d**) 10% donor blood (*v*/*v*) mixed with 20 mM dipeptiven.

Donor pRBCs were added to recipient-washed platelets to form 1%, 5%, or 10% (*v*/*v*) mixtures at 37 °C for 5 min. We also treated 10 % (*v*/*v*) mixtures with glutamine (20 mM) or dipeptiven (20 mM) within 1 min to examine whether CD40L expression can be inhibited at 37 °C for 5 min. FITC-labeled mouse IgG1κ and PE-labeled mouse IgG1κ served as background controls. To measure CD40L expression, samples were stained with a saturating concentration of anti-CD40L-FITC and anti-CD41a-PE monoclonal antibodies at approximately 22–28 °C in the dark for 20 min. Individual platelets were identified by anti-CD41a-PE immunofluorescence and visualized using a logarithmic scale dot plot and side scatter (Figure 2).

### 2.6. Flow Cytometry

The FACSCalibur flow cytometer (BD, Franklin Lakes, NJ, USA), a standard two-color filter configuration, and CellQuest cell analysis software (BD, Franklin Lakes, NJ, USA) were used to determine platelet DCF fluorescence generation and CD40L expression in pRBCs and washed platelet mixed samples. Data from 10,000 platelets were collected for each sample.

### 2.7. Statistical Analysis

Demographic data were presented as mean ± standard deviation (Table 1 and Table 2). Statistical significance for the ROS generation and CD40L expression levels was determined using one-way analysis of variance (ANOVA) with the Bonferroni post hoc test. SPSS software version 20 (IBM Corp, Armonk, NY, USA) was used for all analyses. A *p* value of <0.05 was considered statistically significant. Additionally, with the use of G*Power software (version 3.1.3, Franz Faul, Universität Kiel, Germany), a power analysis performed by the one-way ANOVA. After ordering α = 0.05, *n* = 22, then we calculated the power (1 − β) = 12.8%, 8.4%, and 10.3% among controls with 1–10% of pRBCs and washed platelets mixture, 10% of pRBCs and washed platelets mixture with glutamine, and 10% of pRBCs and washed platelets mixture with dipeptiven, respectively for DCF fluorescence generation. Moreover, we ordered α = 0.05, *n* = 13, we calculated the power (1 − β) = 98.7%, 14.2%, and 12.6% among controls with 1–10% of pRBCs and washed platelets mixture, 10% of pRBCs and washed platelets mixture with glutamine, and 10% of pRBCs and washed platelets mixture with dipeptiven, respectively, for CD40L expression.

## 3. Results

**Figure 3:**
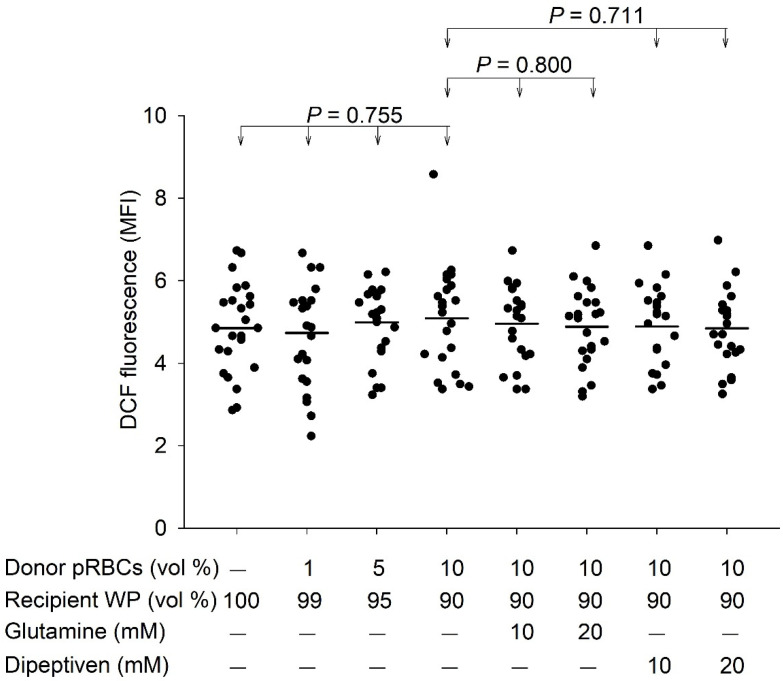
DCF fluorescence generation after the mixing of pRBCs and washed platelets and response to antioxidant treatment (*n* = 22). MFI, mean fluorescence intensity; pRBCs, packed red blood cells; WP, washed platelets; vol, volume. Mean values are indicated by horizontal lines.

**Figure 4:**
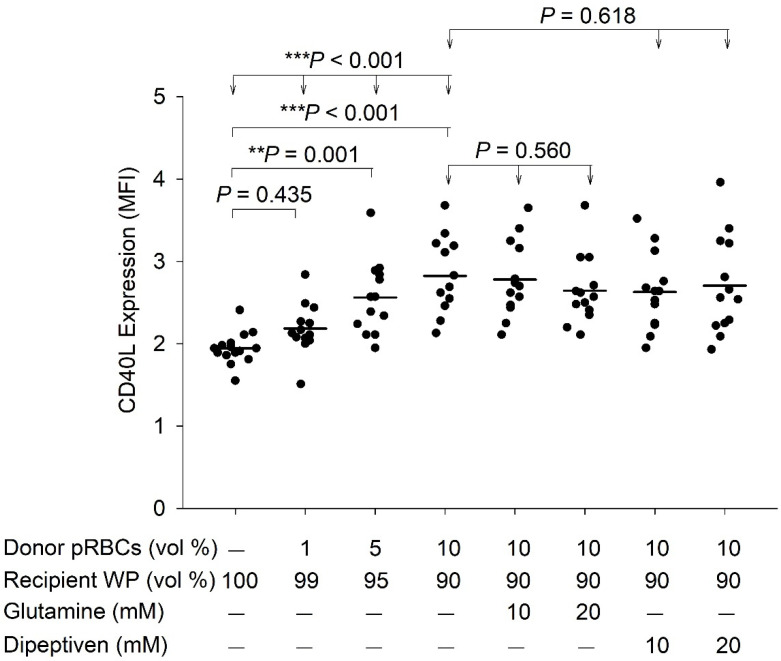
CD40L expression after the mixing of pRBCs and washed platelets and response to antioxidant treatment (*n* = 13). MFI, mean fluorescence intensity; pRBCs, packed red blood cells; WP, washed platelets; vol, volume. Mean values are indicated by horizontal lines.; ** *p* < 0.01; *** *p* < 0.001.

Table 1 and Table 2 summarizes the demographic characteristics of the patients. The mixing of pRBCs and washed platelets revealed no significant effect in ROS generation, as determined by DCF fluorescence (*p* > 0.755). Furthermore, at 10% pRBCs and washed platelets mixture, the DCF fluorescence levels revealed no significant difference after treatment with glutamine (*p* = 0.800) or dipeptiven (*p* = 0.711) (Figure 3). In contrast, pRBCs and washed platelets mixture significantly induced platelet CD40L expression in a concentration-dependent manner (*p* < 0.001). CD40L expression increased significantly at 1% (*p* = 0.435), 5% (*p* = 0.001), and 10% (*p* < 0.001) mixture ratios. However, at 10% pRBCs and washed platelets mixture, the CD40L expression remained unchanged after treatment with glutamine (*p* = 0.560) or dipeptiven (*p* = 0.618) (Figure 4).

## 4. Discussion

We examined the causal relationship among mixing pRBCs and washed platelets, platelet ROS levels, and platelet CD40L expression. Our results demonstrated that mixing pRBCs and washed platelets does not significantly affect platelet ROS generation; nevertheless, this approach has a significant potential to increase CD40L expression, which has been implicated in transfusion reactions [4,16,18,19,20]. Next, we used antioxidants to antagonize ROS and further assess whether mixing pRBCs and washed platelets could effectively prevent ROS generation and reduce CD40L expression. Thus, if the pRBCs and washed platelet mixture affects ROS, which is also expected to be affected by the antioxidant as ROS and the antioxidant are complementary factors, we hypothesized that the mixture would induce ROS generation. However, our result demonstrated that ROS was not inhibited by antioxidants and hence was not affected by pRBCs and washed platelet mixture, contradicting our hypothesis (Figure 3) and indirectly confirming that this approach cannot induce ROS generation. Second, we hypothesized that the pRBCs and washed platelet mixture effect on CD40L expression could be mediated by ROS. To examine the effect of ROS generation on CD40L expression theoretically, the blood mixture should be treated with a ROS-generating compound; however, no ROS-generating compound was available. Therefore, it was difficult to demonstrate the effect of ROS on CD40L expression directly. To overcome this limitation, we indirectly tested this hypothesis using antioxidants. Our result revealed that CD40L expression was not inhibited by antioxidants, contradicting our hypothesis (Figure 4). Therefore, it was indirectly confirmed that ROS generation cannot induce CD40L expression in platelets. Taken together, we demonstrated that ROS generation is not an upstream master switch for platelet CD40L expression. In other words, the induction of platelet CD40L expression by the mixing pRBCs and washed platelets did not occur through platelet ROS generation.

If the causality between platelet ROS generation and CD40L levels is true, one or more of the three factors (i.e., platelet activation, ROS generation, and CD40L expression) could be a therapeutic target to prevent the incidence of transfusion reaction. The first target is the platelet itself, which can be observed in chronic antiplatelet agents, administered for diseases, such as coronary artery disease, ischemic stroke, peripheral artery occlusion disease, arrhythmia, and deep venous thrombosis [21,22]. However, blood transfusion is often performed due to hemorrhage and surgery. Both conditions include the corresponding anesthesia, especially neuraxial blockade, which is required for the surgery and may contraindicate the use of antiplatelet agents [21,23]. The second target is platelet ROS generation. Antioxidants was readily available [24], as they are naturally present in food, and their demand has increased rapidly. They have also been suggested as ingredients in health-promoting foods. These peptides are encrypted from various food-derived protein sources by chemical and enzymatic hydrolysis and microbial fermentation [24]. In addition, glutamine and dipeptiven have been commercialized and can be administrated via the enteral or intravenous route [25,26]. Therefore, antioxidant targeting of ROS could increase the accessibility of clinical treatment agents and reduce the cost. The third target is platelet CD40L. Although we observed that mixing pRBCs and washed platelets induces CD40L expression, a CD40L antagonist is currently unavailable. Above all, we expect that if the upstream (ROS) response is controlled, the downstream (CD40L) response can be suppressed; however, the causal relationship among ROS, CD40L, and antioxidants is weak or even nonexistent, and this is an unexpected but useful finding to avoid errors in future research.

We observed that mixing pRBCs and washed platelets does not affect platelet ROS generation, which contrasted with the findings of previous studies on ROS-related transfusion reactions in other circulatory cells and endothelium. First, Zeeuw et al. reported that macrophages produce ROS, which damage the pulmonary endothelium and causes transfusion-related acute lung injury [13]. Second, Semple et al. reported that intravenous immunoglobulin prevents murine antibody-mediated acute lung injury through neutrophil ROS production, thus, alleviating tissue damage [10]. Third, Khoy et al. reported that ROS production upon neutrophil activation plays a role in transfusion-related acute lung injury [11]. Fourth, Czubak et al. reported that oxidative stress increased after RBC transfusion, but not after platelet transfusion [12]. Above all, this transfusion-related increase in ROS is only observed in macrophages [13], neutrophils [10,11], and RBCs [12], but not in platelets, which is consistent with the results of our studies. According to our results, which show that mixing pRBCs and washed platelets does not increase platelet ROS levels, one can imagine that the administration of antioxidants does not suppress the platelet ROS level that is not increasing, as noted in our results (Figure 3). Therefore, the effect of antioxidants on platelet ROS-related transfusion reaction should not be overemphasized.

After mixing pRBCs and washed platelets, the nature of platelet ROS generation must be presented by an accurate experiment without exaggerating the pRBCs and washed platelets mixture-related causal effect between ROS and CD40L, even though ROS have been known to cause transfusion reactions in other circulatory cells [3,10,11,13,14,15] and have the potential of a double-edged sword in physiology and pathology [27]. ROS have crucial roles in normal physiological processes, such as redox regulation of protein phosphorylation, ion channels, and transcription factors. They are also required for biosynthetic processes, including thyroid hormone production and crosslinking of extracellular matrix [9]. In contrast, the following are the disadvantages of ROS: first, ROS are considered detrimental to the overall health of the organism [8,9,28]. Second, aberrant production or regulation of ROS activity reportedly contributes to the development of some prevalent diseases and conditions, including cancer and cardiovascular disease. However, our results implicated that these disadvantages of platelet ROS may not include transfusion reactions.

For clinical application, we used both glutamine and dipeptiven (stable glutamine) as antioxidants, which are commonly used as dietary supplementation via enteral or parenteral nutrition [25,26], according to the European Society for Clinical Nutrition and Metabolism guideline on clinical nutrition in the intensive care unit [29]. Patients in the intensive care unit often need pRBC transfusion. Glutamine is an essential amino acid required by virtually all mammals. However, glutamine is unstable and can be degraded spontaneously in solution, which might also have occurred in the mixing of pRBCs and washed platelets sample in our study. Therefore, we further chose dipeptiven, which is stable in an aqueous solution and does not degrade spontaneously. Dipeptiven metabolizes into glutamine and alanine. At 22–28 °C with a pressure of 1 atm, dipeptiven has 10-times more soluble than glutamine. Additionally, glutamine does not withstand sterilization procedures, unlike dipeptiven. The high solubility of dipeptiven makes it suitable for parenteral nutrition. Glutamine has an antioxidative effect secondary to its role in glutathione synthesis. It can increase the enzyme activity of glutathione peroxidase and reduce ROS production, therefore increasing the total antioxidant level [30]. Antioxidants in a physiological setting prevent an increase in ROS concentration that may cause body damage. In normal physiological processes, antioxidants affect signal transduction, regulation of proliferation, and immune response. However, it did not showed a pRBCs and washed platelets mixture-related significant effect on platelet ROS levels in the present study, suggesting that antioxidants have neither encouraging nor preventive effects on ROS generation in the pRBCs and washed platelet mixture.

We observed that mixing pRBCs and washed platelets induces platelet CD40L expression. This result may be explained by the presence of small amounts of leukocytes, platelets, and residual plasma in the pRBC samples used [13]. The residual plasma contains platelet agonists, including ADP, thromboxane A2, collagen, and thrombin [14]. Hence, after the blood components were mixed, recipient washed platelets were stimulated by donor leukocytes, activated platelets, and various platelet agonists from the pRBCs, which in turn activated that the washed platelets. This process would promote the transport of CD40L within α-granules to the platelet surface to function as a membrane-bound CD40L and to be then secreted as a soluble CD40L [15]. CD40L expression has occurred in transfusion reactions as per the following previous studies: first, platelets cause transfusion-related acute lung injury via the CD40/CD40L complex [17]. Second, circulatory CD40L can be cleaved from the membrane-bound CD40L of platelets and has been associated with transfusion-related acute lung injury [20]. Third, platelet CD40L may induce transfusion-related acute lung injury [20] through the activation of leukocytes and endothelial cells, and cytokine release [4]. This CD40/CD40L complex may be a major target in a transfusion-related acute lung injury prevention strategy [4,17,20].

In this study, we demonstrated that mixing pRBCs and washed platelets does not affect CD40L expression via platelet ROS generation; therefore, antioxidants are unable to reduce CD40L expression. Our finding contrasted with previous reports of a direct link between ROS and increased platelet CD40L expression. First, ROS are known to play an important role in human platelet CD40L expression under physiological, pathological, and inflammatory conditions [5,6,7]. Moreover, ROS-mediated platelet CD40L upregulation is observed in patients with hypercholesterolemia [7]. Therefore, even though ROS have the potential to increase CD40L in blood components, as observed in previous reports, CD40L expression may not be related to platelet ROS in pRBCs and washed platelet mixtures. This observation suggests that to reduce the incidence of transfusion reactions by inhibiting CD40L expression, ROS generation in platelets should not serve as a therapeutic target.

There are three strengths of this study. First, this is a leading study because it is unknown whether transfusion reactions are mediated via ROS and/or CD40L in a causal manner in platelets, and we explored the causality relationships between mixing pRBCs and washed platelets, ROS generation, CD40L expression, and transfusion reactions with exclusive focus on platelets. Second, we used indirect approaches to demonstrate that pRBCs and washed platelets mixtures do not induce platelet ROS generation and that platelet ROS may not contribute for platelet CD40L expression, as confirmed by the antioxidant treatment. Third, we are willing to publish some unexpected results. Our finding that indicated no response to pRBCs and washed platelet mixture, such as platelet ROS generation, is a valuable component of the scientific literature since it directs us toward unabridged science. This will save the time of other researchers, who may pursue research by repeating our findings.

There are three limitations in this study. First, the mixing of blood in vitro was not similar to blood transfusion in vivo. However, this type of study is hard to conduct, and it can be complicated to control the confounding factors among ROS and CD40L expression within platelets in vivo. In vitro research still has some advantages over in vivo research, such as (1) tight control of the physical environment, (2) reduced cost, (3) higher throughput, and (4) reduced ethical considerations. Second, owing the low stability and short half-life of ROS, the use of other method(s) should be considered to validate the present findings. Proteins represent a wide target for ROS, therefore protein oxidation in platelets may be useful for determining markers of oxidative stress (ROS generation) [31]. Third, our platelet samples were obtained from patients scheduled for cardiac surgery; thus, extrapolation of our results to other patient populations should be done with caution.

## 5. Conclusions

This study is novel since it evaluates the causal relationship among pRBCs and washed platelets mixture and ROS- and CD40L-related transfusion reactions occurring in platelets. We expected that if the causality was true, ROS should be considered an upstream targets with antioxidants being valuable and accessible therapeutic agents, and platelet CD40L should be regarded as targets to reduce the occurrence of transfusion reactions. However, platelet ROS and CD40L did not present such causal relationship in platelets. Indeed, our study demonstrated an increase in platelet CD40L expression, which was independent of platelet ROS. ROS have been highlighted as having versatility through cellular or molecular aspects in a transfusion reaction. However, excluding the mixing of pRBCs and washed platelets from platelet ROS generation causes a transfusion reaction. We observed that platelet CD40L can be a potential therapeutic target. The comprehensive evaluation of transfusion risk factors, management, target on circulatory cells other than platelets or components, and prevention of transfusion-related acute lung injury through antioxidant administration before transfusion, should be further studied.

## Figures and Tables

**Table 1:** Demographic characteristics of patients for detection of ROS.

	Value
No. of cases	22
Age (years)	63.8 ± 9.5
Height (cm)	165.3 ± 8.8
Weight (kg)	66.8 ± 7.8
BMI (kg/m^2^)	24.6 ± 3.7
Women/Men	8/14

Data are presented as mean ± standard deviation. BMI, body mass index.

**Table 2:** Demographic characteristics of patients for detection of CD40L.

	Value
No. of cases	13
Age (years)	62.2 ± 13.1
Height (cm)	165.3 ± 11.0
Weight (kg)	66.2 ± 8.6
BMI (kg/m^2^)	24.5 ± 4.3
Women/Men	5/8

Data are presented as mean ± standard deviation. BMI, body mass index.

## Data Availability

The datasets generated and/or analyzed during this study are available in the Mendeley Data repository, [https://data.mendeley.com/datasets/4fhvtmjsgx/1]. Accessed date: 15 April 2022.

## Supplementary Materials

The following are available online at https://www.mdpi.com/article/10.3390/antiox11061108/s1, Figure S1: Key steps of general anesthesia and cardiac surgery in this study. First, essential hemodynamic monitoring. Second, cross-matched red blood cells can be sent to operation room. Third, arterial catheterization. Fourth, obtain recipient whole blood samples by arterial catheterization. Fifth, anesthesia administration and induction. Sixth, skin incision and operation. Blood sampling before anesthesia induction and skin incision and operation can theoretically eliminate confounding factors caused by anesthetics and tissue injury-related factors in the blood circulatory system. The rationale for specifically using blood from recipients undergoing cardiac surgery was described in supplemental materials (Supplemental Figures S1 and S2).
