# Supplementary material for: Platelet CD40L Expression Response to Mixing of pRBCs and Washed Platelets but no Causality Association between Platelet ROS Generation and CD40L Expression: An In Vitro Study
Source: Antioxidants (Basel). 2022 Jun 2;11(6):1108. doi: 10.3390/antiox11061108 (PMC9219937; doi:10.3390/antiox11061108)
Supplement: Supplementary file 1 [file antioxidants-11-01108-s001.zip › antioxidants-1707670-supplementary.pdf]

Supplemental materials

Rationale for specifically using blood from recipients undergoing cardiac surgery

Healthy volunteers were the first choice; however, they have no cross-matched blood. To prevent repetition and inadequate explanation, we did not discuss this issue in this manuscript. In summary, first, cross-matched red blood cells were always available, and they were sent to the operating room from the blood bank before anesthesia, enabling us to obtain donor cells. Second, patients undergoing cardiac surgery require arterial catheterization for aggressive hemodynamic monitoring. The arterial catheterization approach used for recipient blood sampling before anesthesia and skin incision prevents contamination from anesthetics and tissue damage that may be a confounding factor affecting platelet function and activation (Figure S1). In addition, there were adequate cardiac surgery case numbers.

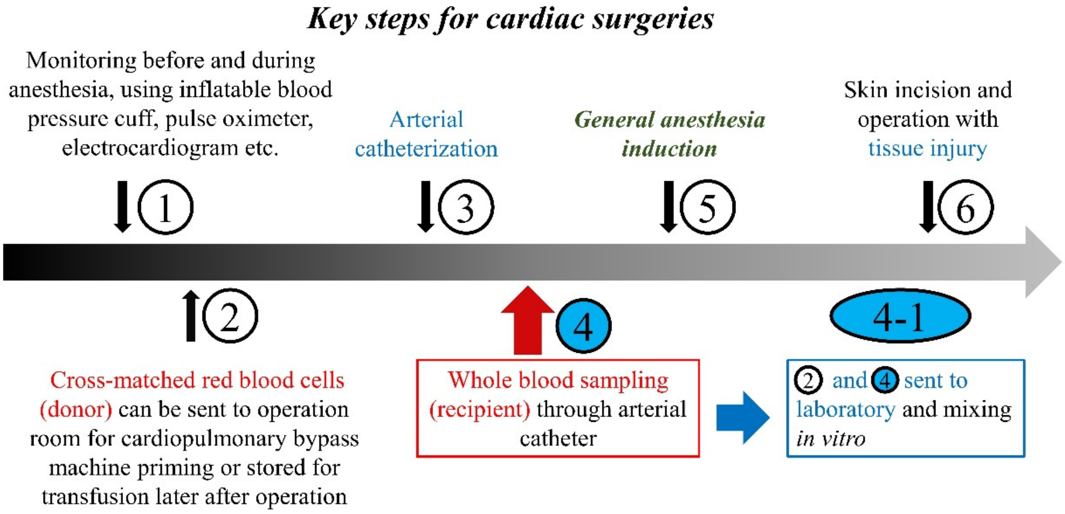

Figure S1. Key steps of general anesthesia and cardiac surgery in this study. First, essential hemodynamic monitoring. Second, cross-matched red blood cells can be sent to the operating room. Third, arterial catheterization. Fourth, obtain recipient whole blood samples by arterial catheterization. Fifth, anesthesia administration and induction. Sixth, skin incision and operation. Blood sampling before anesthesia induction and skin incision, and operation can theoretically eliminate confounding factors caused by anesthetics and tissue injury-related factors in the blood circulatory system.

What if we chose other various types of major surgeries?

In various types of major surgeries (great vessel surgery, thoracic surgery, exploratory laparotomy, large hepatic tumor resection, multiple trauma, removal of large tumor, pelvic fracture, long bone open fracture, among others), patients are usually elderly and many of them also take antiplatelet agents. If these patients were to be excluded, the case number would be very low, whereas if they were included, the heterogeneity of the patients would increase. By contrast, cardiac surgery is almost always performed with the use of antiplatelet agents and the heterogeneity among patients is low. As for whether antiplatelet agents will affect the performance of CD40L expression, it seems that not enough antiplatelet effect occurred, as our experimental data demonstrated that CD40L expression increased in a dose-response manner after pRBCs and washed platelets were mixed.

Additional disadvantages from other major surgeries, and therefore reasons they were not chosen:

1. Various types of tissue injury with various recipient blood contamination that lead to various baseline platelet activation;
2. Confounding factors of tissue injury and anesthetic agents;
3. Various patient ages;
4. Halfway through the operation, if there was heavy bleeding, and we hurriedly decided to receive pRBCs from the blood bank for transfusion and conduct the experiment at the same time, this would cause the clinical and experimental steps to be unprepared, thereby endangering the safety of the patients and impairing the quality of the experiment;
5. Insufficient case numbers;
6. Transfusion and preparation of cross-matched red blood cells before surgery were not conducted routinely.

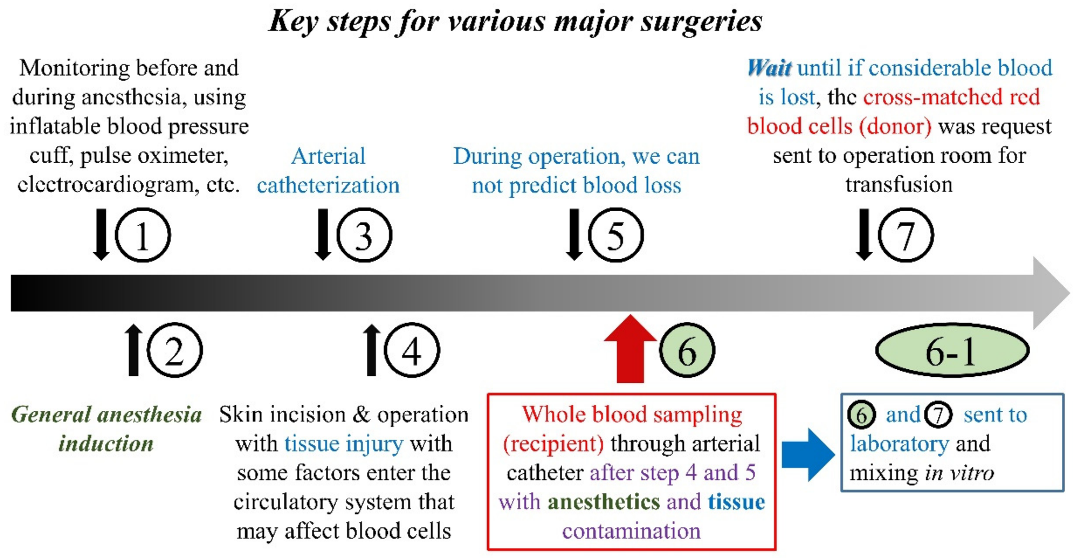

Figure S2. Key steps of general anesthesia and various major surgeries.

This figure illustrates which other types of major surgeries were not selected because they do not always require transfusion and the same type of surgery has varying possibilities of cross-matched red cell transfusion. The request for prepared cross-matched red blood cells is often dependent on massive blood loss, after which red blood cell transfusion is performed. By this time, the operation has caused major tissue damage, and the circulation blood sample is often contaminated with anesthetic agents, and operation- and tissue damage-related circulation factors. This may be a confounding factor in the platelet function test.
